# Morphological and Mechanical Properties of the Human Patella Tendon in Adult Males With Achondroplasia

**DOI:** 10.3389/fphys.2018.00867

**Published:** 2018-07-20

**Authors:** David T. Sims, Gladys L. Onambélé-Pearson, Adrian Burden, Carl Payton, Christopher I. Morse

**Affiliations:** Health, Exercise and Active Living Research, Manchester Metropolitan University, Manchester, United Kingdom

**Keywords:** Achondroplasia, patella tendon, stress, strain, Young's Modulus

## Abstract

Achondroplasia is a genetic mutation of fibroblast growth factor receptor resulting in impaired growth plate development in long bones due to lower collagen turnover. Despite the characteristic shorter stature and lower strength in Achondroplasic groups, little is known of the tendon mechanical properties under loading. The aim of this study was therefore to conduct a between measure design of patella tendon (PT) mechanical properties (stress, strain, stiffness and Young's Modulus) in 10 men with Achondroplasia (22 ± 3 years) and 17 male controls (22 ± 2 years). PT mechanical properties were measured during isometric maximal voluntary contraction (iMVC) of the knee extensors using ultrasonography. The Achondroplasic group produced 54% less stress at iMVC than controls (29.4 ± 8.0 v 64.5 ± 14.0 MPa, *P* < 0.001, *d* = 3.12). Maximal excursion of the Achondroplasic PT was 22% less than controls at iMVC (7.4 ± 2.1 v 5.5 ± 1.7 mm, *P* < 0.001, *d* = 0.99), but there was no difference in strain between groups (13 ± 4 v 13 ± 3%, *P* > 0.05). Achondroplasic PT were 47% less stiff (748 ± 93 v 1418 ± 101 N·mm^−1^, *P* < 0.001, *d* = 6.89) and had a 51% lower Young's modulus (0.39 ± 0.09 v 0.77 ± 0.14 GPa, *P* < 0.001, *d* = 3.46) than controls at iMVC. Achondroplasic PT are indeed more compliant than controls which may contribute to lower relative force production. The causes of higher Achondroplasic PT compliance are unclear but are likely due to the collagen related genetic mutation which causes Achondroplasia.

## Introduction

Achondroplasia is a genetic condition characterized by shorter growth of long bones in the appendicular skeleton relative to the torso (Horton et al., 2007). The genetic mutation occurs within the fibroblast growth factor receptor 3 (FGFR3) which is part of a family of fibroblasts that are essential for the development, repair and turnover of collagen (Benjamin and Ralphs, 2000). The Achondroplasic condition is synonymous with impaired bone growth and development, but given that the human tendon is predominantly made up of collagen fibrils (Wang, 2006), it is possible that the altered FGFR3 may impact intrinsic properties of the Achondroplasic tendon compared to age matched averaged statured individuals (controls). However, Achondroplasic tendon compliance (or joint laxity) have only been commented on but not quantified (Bober et al., 2008; Akyol et al., 2015).

The human tendon is a collagenous, viscoelastic material which deforms in a curvilinear fashion when loaded (Maganaris and Paul, 2000; Reeves, 2006; Wang, 2006; Onambélé et al., 2007). Both the force through the cross-sectional area (CSA) of tendon (here as stress) and the amount of relative lengthening of the tendon during loading (here as strain) contribute to the tendon's role in transferring force from muscle to bone. The ratio of stress to strain provide Young's Modulus, which implies the tensile strength of the tendon whilst accounting for morphological properties of CSA and tendon length. A tendon that lengthens more for a given force production is more compliant and less effective at translating force to the bone and influences power output by up to 40% (Galantis and Woledge, 2003). This can lead to a higher oxygen cost during activities such as walking and running (Fletcher et al., 2010), which has recently been observed in a male Achondroplasic population (Sims et al., 2018). Whilst tendon loading and excursion during contraction has been described for differing human populations, both *in vivo* and *in vitro*, there remains no data for Achondroplasic populations.

Assuming tendon collagen is unaffected in Achondroplasic populations, there are likely to be differences in tendon morphology compared to controls due to stature differences between groups. The likely differences in tendon morphology between groups are also not likely to scale to anthropometric measures. For example, there is a downscaling of patella tendon morphology (CSA and length) from adults to children (O'Brien et al., 2010b). Given that the Achondroplasic limb length is disproportionate to the torso, compared to controls, a similar downscaling of patella tendon morphology may not be apparent. During contraction, differences in tendon morphology affect the rate of force transduction to the bone (Reeves, 2006). We previously observed that absolute and relative knee extensor (KE) force production is lower in Achondroplasic adult males to controls (Sims et al., 2017), and the likely patella tendon morphology differences between groups may explain some of the difference. There appears to be however, no measure of *in vivo* morphological or mechanical properties of the Achondroplasic tendon to make this assumption.

The aim of this study was therefore to measure the *in vivo* morphological properties of Achondroplasic patella tendons and compare them to controls. Furthermore, we aimed to measure the mechanical properties of the patella tendon during isometric maximal voluntary contraction (iMVC) of the KE and to observe the contractile properties of the vastus lateralis (VL). Young's Modulus would be calculated to appropriately scale Achondroplasic tendon properties and identify tensile strength of the patella tendon for both groups. The hypotheses of this study were that the Achondroplasic group would produce less KE force, have smaller dimensions of the patella tendon and in turn have more compliant patella tendons than controls.

## Methods

### Participants

After written, informed consent, 27 participants free from lower limb injury and of good health volunteered to participate in the study [mean (SD): 10 adult males with Achondroplasia, age: 22 (3) years, mass: 61.8 (8.5) kg, stature: 137.8 (4.7) cm, and 17 adult males, age: 22 (2) years, mass: 78.3 (10.7) kg, stature: 178.7 (8.3) cm]. Ethical approval was obtained from the local committee (Manchester Metropolitan University) and each participant attended one testing session at the laboratories of Manchester Metropolitan University where architectural properties of the patella tendon and vastus lateralis (VL) were measured, followed by isometric maximal voluntary contractions (iMVC) measurements of the KE. The study was a cross-sectional, between subject design which was developed in line with the Strengthening the Reporting of Observational Studies in Epidemiology (STROBE) statement.

### Patella tendon cross sectional area and volume

**Figure 1:**
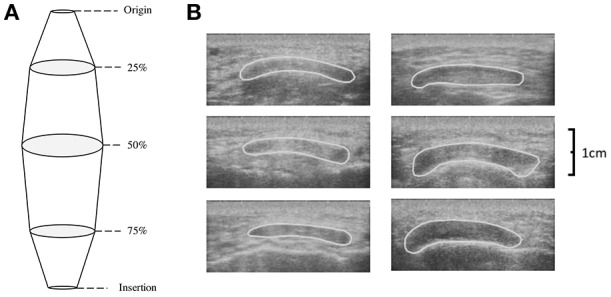
**(A)** Schematic depicting the calculation of tendon volume using the truncated cone method and **(B)** transverse ultrasound scans of the patella tendon CSA at 25% (top), 50% (middle), and 75% (bottom) of patella tendon length for a typical Achondroplasia (Left) and control (Right) control participant.

While at rest participants sat in an isokinetic dynamometer with their dominant leg strapped into the lever arm so that 90° of knee flexion was attained (180° = full extension). Patella tendon origin and insertion were identified using ultrasonography (Technos MXP Biosound Esaote, UK) by holding a 5 cm 7.5 MHz linear array probe in the sagittal plane to the patella tendon. Intervals of tendon length (25, 50, and 75%) were measured and marked. CSA of the patella tendon (CSA_PT_) was taken by applying water-soluble transmission gel to the probe and placing along the sagittal plane of the patella tendon at the marked percentages of patella length with minimal pressure. Depth of view was such that medial and lateral borders of the patella tendon were viewable. Image recordings were AVI format at a sample frequency of 25 Hz; single images were selected using capture software (Adobe Premiere Elements version 10, Adobe Systems) and analyzed using digitizing software (NIH ImageJ, Version 1.44o, National Institutes of Health, Bethesda, Maryland). High reliability has been shown measuring patella tendon length (Skou and Aalkjaer, 2013) and CSA_PT_ with ultrasound (Gellhorn and Carlson, 2013). Previous data on the reliability of the CSA_PT_ using ultrasound is reported between 0.870 and 0.990 (Reeves et al., 2003b; Onambélé et al., 2007; Burgess et al., 2009; Gellhorn and Carlson, 2013). To confirm this within our study, CSA_PT_ was measured on a single occasion and digitized on two separate occasions to determine intrascan reliability and coefficient of variation (CV). Patella tendon volume was then calculated using each individual CSA_PT_ within the truncated cone method (Figure 1):

$$\begin{array}{l} \text{ R }=\;\sqrt{\frac{\text{ A }}{\pi }}\;\\ \text{ TCV }=\frac{\pi }{3}\;.\;\left({\text{L}}_{\text{PT}}\;\cdot \;({\text{R}}_{1}^{2}\;+\;{\text{R}}_{2}^{2}\;\cdot \;{\text{R}}_{2})\right))\\ \text{ CV }=\;\frac{\left(\text{A }\times \;{\text{L}}_{\text{PT}}\right)}{3}\\ \text{Tendon Volume }=\text{C}{\text{V}}_{\text{prox}}\;+\text{ TC}{\text{V}}_{1}\;+\text{ TC}{\text{V}}_{2}\;+\text{ C}{\text{V}}_{\text{dist}} \end{array}$$

Where R is the radius (cm) of a given scan, A is the measured CSA_PT_ (cm^2^), TCV is the mid-proximal (TCV_1_) and mid-distal (TCV_2_) truncated cone volumes (cm^3^) representing 25–50% and 50–75% of the tendon respectively, L_PT_ is patella tendon length (cm) between two measured points, R_1_ and R_2_ are the radii (cm) of two scans respectively, while CV is cone volume (cm^3^) of the proximal (CV_prox_) and distal cone (CV_dist_) representing 0–25% and 75–100% of the tendon respectively. Tendon volume (cm^3^) is the sum of all inter-scan volumes.

### Knee extensor torque measurements

Torque production during KE iMVC (KE iMVCτ) of the dominant leg (Achondroplasia *N* = 9/10 right leg, control *N* = 16/18 right leg) was recorded using an isokinetic dynamometer (Cybex Norm, Cybex International Inc., NY, USA). Firstly, a counter weight was fixed to the dynamometer to minimize and standardize the compliance of the device. Participants were seated upright with the dynamometer and chair positioned in accordance with the calibration guidelines, so the lateral epicondyle was aligned with the dynamometer's central axis of rotation. Particularly in the Achondroplasic group, the chair and dynamometer were adjusted to align the medial malleolus if needed. Additional padding was placed behind the spine to stabilize the individual to ensure the knee maintained the set position throughout contractions. Velcro straps were used to secure the dominant leg to the chair via the distal portion of the thigh, while the lever arm was attached to the tibia at ~80% of its length. Participants warmed up by performing six continuous submaximal concentric contractions of the KE and knee flexors (KF). To reduce the effect of creep and to ensure preconditioning of the patella tendon (Maganaris, 2003; Pearson et al., 2007), participants completed four KE iMVCs at 90°Flexion (180° = full extension) with ~120 s rest between trials. Following warm up, two KE iMVC trials were recorded with participants verbally encouraged to exert as much force as possible. A ramped iMVC, lasting ~5 s, was instructed with visual feedback provided to all participants. KE iMVCτ was assumed based upon a visible plateau on a monitor and were recorded (2,000 Hz) on a computer (iMac, California) using an acquisition system (AcqKnowledge, Biopac Systems, California).

### VL architecture and fascicle displacement

The origin and insertion of the VL were identified using B-mode ultrasonography (Technos MXP Biosound Esaote) by holding a 5 cm 7.5 MHz linear array probe along the transverse plane of the muscle. Upon identification of landmarks, a tape measure was used to determine VL length. *In vivo* muscle architecture of the VL was measured using B-mode ultrasonography during the last warm up KE iMVC to observe fascicle length (cm) and pennation angle (θ). Both fascicle length and pennation were measured during rest and KE iMVC to observe the change in each respective variable. The same linear array probe described above was held on the mid-sagittal plane on a previously established mid-point of the VL; measured equidistant from the origin-insertion and medial-lateral muscular borders. With water-soluble transmission gel, the probe was held normal to the skin with minimal pressure. View depth was set to ensure a number of fasciculi insertion points and deep aponeurosis were in view (Maganaris, 2001). Imaging and torque production were synchronized by an external voltage trigger enabling the accurate attainment of iMVC-to-ultrasound. Image recordings were AVI format at a sample frequency of 25 Hz; single images were selected using capture software (Adobe Premiere Elements version 10, Adobe Systems). Images of the VL at rest and iMVC were analyzed using digitizing software (NIH ImageJ, Version 1.44o, National Institutes of Health, Maryland). Fascicle length was determined as the distance between the superficial and deep aponeuroses along a visible fascicle (Maganaris, 2001). Pennation angle was defined as the insertion angle of the fascicle into the deep aponeurosis (Maganaris, 2001). With the VL being one of the larger muscles in the body, invariably the dimensions of the probe were not large enough to capture a full fascicle, for these cases linear extrapolation was used to determine fascicle length as little error (2–7%) is observed at the midpoint of the muscle (Finni et al., 2003), again using digitizing software described above.

### Tendon elongation measurements

**Figure 2:**
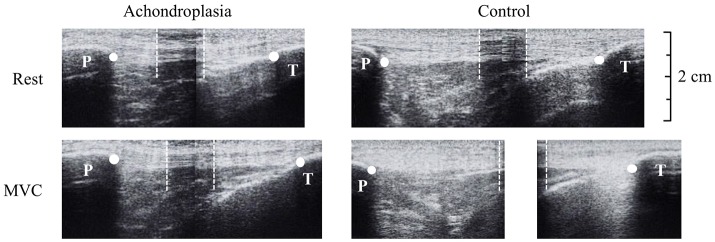
Ultrasound images during tendon elongation measures of the Achondroplasic (left images) and control (right images) patella tendon from rest (top) to maximal voluntary contraction (bottom). White dots represent the measurement of tendon length from patella tendon origin (patella, P) and insertion (tibia, T). For the longer tendons that exceed the ultrasound probe's field of view, an echo-absorptive marker was used as a reference point to help stitch images together (Onambélé et al., 2007). The boundaries of the marker are identified by the white dotted lines.

Tendon elongation was observed from rest to iMVC. 50% of the measured resting tendon length described above was used to place a thin (~10 mm) echo absorbing marker (Micropore tape) on the skin, across the tendon, to act as a reference marker. The ultrasound probe was held in the sagittal plane over the patella tendon so that the reference marker was identifiable with both the patella tendon origin (proximal, trial 1) and the patella tendon insertion (distal, trial 2) in the same image. Ultrasound images were then stitched together using digitizing software (GIMP) and analyzed using Image J. High reliability (*r* = 0.910) of tendon excursion using this method is reported elsewhere (Onambélé et al., 2007). Participants were instructed to perform a ramped iMVC where the test was terminated once a plateau of the torque trace was observed on a monitor. Ultrasound imaging and torque production were synchronized using an external voltage trigger enabling the accurate attainment of iMVC-to-ultrasound. As described by Onambélé et al. (2007), analysis of the images were completed after the calculation of torque at 10% intervals of iMVC, where excursion was determined from the respective origin and insertion of the tendon to the respective superior and inferior edge of the reference marker (Figure 2). At each 10% interval of iMVC, the displacement of the patella tendon from the respective trial 1 and trial 2 measurements added to the width of the tape placed over the patella tendon to measure total tendon length, as described elsewhere (Onambélé et al., 2007). Elongation of the patella tendon was recorded once, but to ensure intrascan reliability, images were digitized twice on separate occasions. These values were then used to determine total strain described below.

### Agonist activation

To accurately measure KE iMVCτ, agonist activation was assessed to estimate the degree of total activation of the KE. To do so, two rubber stimulation pads (size ranging from 70 × 90 to 180 × 100 mm; Uni-Patch, MN, USA) were placed proximally and distally along the transverse plane of the dominant femur. A counter weight was fixed to the dynamometer to minimize the compliance of the device. While in a relaxed state, a percutaneous singlet electrical stimulus (DS7, Digitimer stimulator, Welwyn, Garden City, UK) was passed through the KE at increasing increments (~50 mV) and regular intervals (~20 s) until a plateau of twitch torque was measured. A supramaximal doublet stimulus was subsequently applied to the participant's KE (interstimulus gap 10 μs and pulse width 50 μs) during KE iMVC. Doublet stimulus has been shown to improve the signal-noise ratio in the assessment of central activation (Kent-Braun and Ng, 1999). A second doublet was applied approximately 5 s after the first stimulus when the muscles were fully relaxed, termed the potentiated doublet (Behm et al., 2001). Agonist activation was calculated using the following equation:

$$\begin{array}{l} \text{Activation }\left(\%\right)\;=\;100\;\cdot \left(1-\;\left(\;\frac{\text{t }-\text{ iMVCτ}}{\text{T }}\right)\right) \end{array}$$

Where; t is the interpolated doublet amplitude of the twitch torque, iMVCτ is the isometric maximal voluntary contraction torque and T is the potentiated doublet amplitude (Behm et al., 2001).

### Measurement of coactivation

Co-activation of the KF was measured in all participants during a KE iMVC, and subsequent KF iMVCτ produced at the angle at which peak KE iMVCτ was measured. In order to determine coactivation of the KF, surface electromyography (EMG) was recorded over the biceps femoris (BF) as it is the largest of the KF group, and is representative of the KF group as a whole (Kellis and Unnithan, 1999). Furthermore, surface EMG was deemed adequate despite the adiposity levels in Achondroplasic individuals (Sims et al., 2017), as no differences in EMG readings are observed between groups of differing adiposity (De Vito et al., 2003). Boundaries of the BF were determined using ultrasonography (Technos MXP Biosound Esaote) to ensure consistent placement of EMG electrodes over the KF. When established two pre-gelled, unipolar, 10 mm, Ag-AgCl percutaneous EMG electrodes (Ambu Neuroline 720, Baltorpbakken, Denmark) were placed distally at ~1/3 of muscle length, to avoid the motor unit of the BF, and ~2 mm apart along the mid-sagittal plane of the muscle (NORAXON, Arizona, USA). A third electrode was placed on the lateral epicondyle of the same femur as a reference. Prior to the placement of the electrodes, areas of the skin were shaved, then cleaned using an alcoholic wipe to minimize skin impedance and improve the EMG signal. Raw EMG data were recorded at 2,000 Hz, with a high and low band-pass filter set at 10 and 500 Hz respectively, and a notch set at 50 Hz. The integral of the root mean square was recorded 0.5 seconds either side of the KE and KF iMVCτ to quantify the level of KF muscle coactivation. Based on a linear relationship occurring between torque and EMG activity (Maganaris et al., 1998), KF torque (KFτ) during KE iMVC was derived by converting the percentage activation of KF EMG during KE iMVC to KF EMG during KF iMVC.

$$KF\tau \;=\left(\frac{(\;(KE\;÷\;KF\;)\;\cdot \;100\;)\;}{100}\right)\;\cdot \;KF\;iMVC\text{τ}$$

Where KFτ is the KF torque during KE (N·m), KE is the agonist EMG (mV) recorded of the KE during KE iMVC, KF is the antagonist EMG (mV) recorded of the KE during KE iMVC and KF iMVCτ is the torque (N·m) observed during KF iMVC.

### Net knee extensor iMVCτ

The measurement of agonist and antagonist muscle activation are required for the accurate quantification of net KE iMVCτ production which is used in the calculation of patella tendon force (F_PT_). Net KE iMVCτ was subsequently calculated as the sum of KE iMVCτ and KFτ (Reeves et al., 2003b; Onambélé and Degens, 2006; Onambélé et al., 2007; Pearson et al., 2007; Seynnes et al., 2009).

### Moment arm

A duel-energy X-ray absorptiometry (DEXA) scan (Hologic Discovery, Vertec Scientific Ltd, UK) was used to obtain moment arm length of the patellar tendon (Erskine et al., 2014). Participants were asked to lie on their side in a relaxed state with the dominant knee positioned at 90° using a manual goniometer. A sagittal plane scan was taken of the knee using a 22.3 × 13.7 cm field of view. Obtained scans were exported to, and analyzed on, a Dicom viewer (OsiriZ 5.0.2, Pixmeo Sarl, Geneva, Switzerland). Moment arm length (m) was determined as the perpendicular distance between the estimated tibiofemoral contact point (the estimated joint center) and the patella tendon (Tsaopoulos et al., 2006).

### Calculations of the patella tendon's mechanical properties

To calculate the mechanical properties of the patella tendon, the first measurement of CSA_PT_ and patella tendon elongation, and the average KE iMVCτ was used in both groups.

### Patella tendon force

To calculate the F_PT_, the average net KE iMVCτ achieved between proximal and distal efforts was divided by the patella moment arm length (m).

### Stress and strain

Patella tendon stress was calculated by dividing F_PT_ by CSA_PT_ at mid-tendon length (MPa) while patella tendon strain was the ratio of excursion to resting patella tendon length (%) (Onambélé et al., 2007; O'Brien et al., 2010b).

### Tendon stiffness

Patella tendon force-elongation relationships were fitted with second order polynomial functions forced through zero. Instantaneous patella tendon stiffness values were then calculated at 10% intervals of KE iMVC force (from 10 to 100%), from the gradient of tangential lines along the force-elongation curve (Onambélé et al., 2007).

### Young's modulus

Instantaneous Young's modulus values were calculated as:

$$\text{Young's Modulus }=\;K\;\times \;\left(\frac{L_{PTi}}{CSA_{PT}}\;\right)$$

Where *K* is the calculated stiffness, L_PTi_ is the patella tendon length at each 10% tangential calculation of F_PT_ and CSA_PT_ is the cross-sectional area of the patella tendon at 50% of resting length.

### Standardized measures of tensile properties

With large discrepancies in maximal F_PT_ expected between groups (Sims et al., 2017), the tensile properties of the patella tendon were calculated at the lowest F_PT_ attained by the weakest Achondroplasic participant. Patella stress, strain and Young's Modulus are then presented at this common force level (1756 N) achieved by all participants consistent with previous work (Onambélé et al., 2007; Burgess et al., 2009; Hicks et al., 2017).

### Statistical analysis

All data were collated onto a personal computer (MacBook Pro, California) and analyzed using SPSS (v22.0, IBM). Data were assumed parametric following Shapiro-Wilk and Levene's tests. Repeated measures ANOVA with between group effects were conducted on the CSA_PT_ and VL architecture. Between group comparisons for all measured variables at 10% intervals of iMVC were conducted using independent *t*-tests. Intraclass correlations (ICC) with a one-way random effect model and CV were used for reliability of KE iMVCτ between proximal and distal efforts, and between post scan digitization of CSA_PT_ and patella tendon elongation. Where data violated parametric assumptions, a Mann Whitney-U was performed. Alpha was set at ≤ 0.05 and the power of the study was > 0.80 (G^*^Power) such that effect sizes (Cohen's d and η^2^) are reported; all results are reported as means (SD). All results are reported as means (SD). All inferential statistics at maximal values are reported in the text, while all submaximal values can be found in the online supplementary documentation that accompanies this paper.

## Results

### Anthropometric and reliability measures

There was no difference in age between groups [*t*_(25)_ = 0.658, *P* = 0.522]. The Achondroplasic group were 23% smaller in stature [*t*_(25)_ = 18.95, *P* < 0.001] and 19% lighter in body mass [*t*_(25)_ = 4.598, *P* < 0.001] compared to controls; data are reported in the methods. Reliability of KE iMVCτ between proximal and distal efforts were strong (Achondroplasic group: ICC = 0.968, CV = 3.2%, *P* < 0.001; controls: ICC = 0.932, CV = 3.4%, *P* < 0.001) as were the measures of CSA_PT_ (ICC = 0.965–0.998, CV 1.3–4.4% for all measures in both groups, *P* < 0.001) and patella tendon elongation (ICC = 0.940–0.982, CV 1.5–3.0% for all measures in both groups, *P* < 0.001).

### Architectural properties of the patella tendon and vastus lateralis at rest

The Achondroplasic group had a 32% smaller resting patella tendon length (*P* < 0.001) and a 41% smaller VL than controls (*P* < 0.001, Table 1). ANOVA showed an effect in CSA_PT_ between groups [*F*_(1, 25)_ = 9.803, *P* = 0.004, η^2^ = 28.2], but no effect in CSA_PT_ between each measured interval within groups [*F*_(1.8, 50)_ = 3.299, *P* = 0.051, η^2^ = 11.7], but no interaction effect was found [*F*_(2, 50)_ = 0.142, *P* = 0.868, η^2^ = 0.6]. The Achondroplasic group had a smaller CSA_PT_ at 25% (*P* = 0.013), 50% (*P* = 0.003) and 75% (*P* = 0.010) compared to controls (Table 1). The Achondroplasic patella tendon volume was 48% less than controls (*P* < 0.001). There was no difference between groups' moment arm length (*P* = 0.989, Table 1). The Achondroplasic group did have a 15% longer patella tendon length relative to VL length (*P* = 0.001) and a 42% greater moment arm length to femur ratio, compared to controls (*P* < 0.001, Table 1). There was no difference in the ratio of 50% CSA_PT_ to patella tendon length between groups (*P* = 0.102, Table 1).

**Table 1:** Morphological properties of the patella tendon, vastus lateralis and patella tendon moment arm at rest in Achondroplasia and control.

	**Achondroplasia**	**Control**	***P*-value**	**Test statistic**	**Effect size**	**Mean difference**	**95% CI Lower**	**95% CI Upper**
PT Length (mm)	37.6 (4.3)	55.2 (5.8)	< 0.001	8.007	3.362	17.3	12.8	21.7
VL Length (cm)	19.8 (1.2)	33.6 (1.7)	< 0.001	22.110	9.285	13.9	12.6	15.2
PT length:VL length (%)	19.1 (2.3)	16.2 (1.7)	< 0.001	3.499	1.404	2.8	1.1	4.5
25% CSA_PT_ (mm^2^)	81.6 (8.7)	104.1 (25.4)	0.013	PH	1.314	22.5	5.2	39.7
50% CSA_PT_ (mm^2^)	86.9 (13.8)	110.3 (19.6)	0.003	PH	1.410	23.5	9.0	38.0
75% CSA_PT_ (mm^2^)	79.8 (15.5)	105.1 (25.7)	0.010	PH	1.226	25.3	6.7	43.8
50% CSA_PT_:PT Length (%)	0.45 (0.11)	0.51 (0.08)	0.102	3.635	0.658	2.84	1.23	4.45
PT Volume (cm^3^)	1.72 (0.20)	3.28 (0.92)	< 0.001	5.254	2.785	1.56	0.95	2.17
VL Fascicle Length:VL Length (%)	0.48 (0.13)	0.31 (0.04)	< 0.001	5.328	2.077	32.55	10.64	24.04
Moment Arm (mm)	37.6 (4.3)	37.6 (2.1)	< 0.001	0.014	0.005	0.0	0.0	0.3
Moment arm:Femur length (%)	19.1 (2.8)	11.1 (0.8)	< 0.001	11.440	4.442	8.0	6.5	9.4

*Values displayed as mean (SD)*.

*CI, Confidence Interval; PT, Patella Tendon; VL, Vastus Lateralis; CSA_PT_, patella tendon cross sectional area; PH, Post Hoc*.

### Knee extensor and knee flexor iMVCτ

Adult males with Achondroplasia produced 63% less KE iMVCτ than controls (*P* < 0.001, Table 2). KF iMVCτ was also 82% lower in the Achondroplasic group compared to controls (*P* < 0.001, Table 2). The ratio of KE to KF iMVCτ was higher in the Achondroplasic group compared to controls (*P* < 0.001, Table 2). The ratio between KE iMVCτ and VL length was 39% greater in Achondroplasic group than controls (*P* < 0.001, Table 2).

**Table 2:** Activation and force characteristics of the adult VL in Achondroplasia and controls.

	**Achondroplasia**	**Control**	***P*-value**	**Test statistic**	**Effect size**	**Mean difference**	**95% CI lower**	**95% CI Upper**
KE iMVCt (N·m)	92.8 (5)	260.1 (9.5)	< 0.001	12.83	5.389	160.2	134.5	185.9
KF iMVCt (N·m)	19.0 (7.2)	105.0 (19.2)	< 0.001	13.54	5.815	85.9	72.9	99.0
KE iMVCt:VL Length	0.20 (0.04)	0.13 (0.02)	< 0.001	7.58	2.898	0.08	0.06	0.10
Activation (%)^†^	83.9 (13.9)	92 (5.9)	0.125	2.30	0.831	8.3	-1.9	18.5
Coactivation (%)^†^	42.6 (20)	12.6 (5.3)	< 0.001	5.90	2.356	30.0	19.5	40.5
Net KE iMVCt (N·m)	100.1 (21.7)	273.7 (37.9)	< 0.001	13.39	5.957	166.5	140.9	192.1

Values presented as mean (SD)

KE, knee extensors; KF, knee flexors; iMVCt, isometric maximal voluntary contraction torque; VL, vastus lateralis;

† *Mann Whitney-U*.

### Activation and coactivation

There was no difference in maximal KE activation between the groups (*P* = 0.125), however the Achondroplasic group had a 70% greater coactivation of the BF during KE iMVC compared to controls (*P* < 0.001, Table 2).

### Net knee extensor iMVCτ

Both groups increased KE iMVCτ when corrected for BF coactivation, with the Achondroplasic group increasing by 7% (*P* < 0.001) and controls by 5% (*P* < 0.001), respectively. The net KE iMVCτ produced by the VL was 63% less in Achondroplasic group compared to controls (*P* < 0.001, Table 2).

### Muscle structure and contractile properties

**Figure 3:**
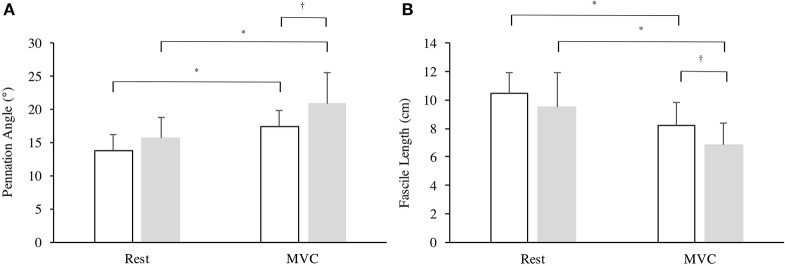
Architectural properties of the vastus lateralis' **(A)** pennation angle and **(B)** fascicle length in Achondroplasia (gray) and controls (white) from rest to iMVC. ^*^ ≤ 0.05, ^†^ ≤ 0.001.

**Figure 4:**
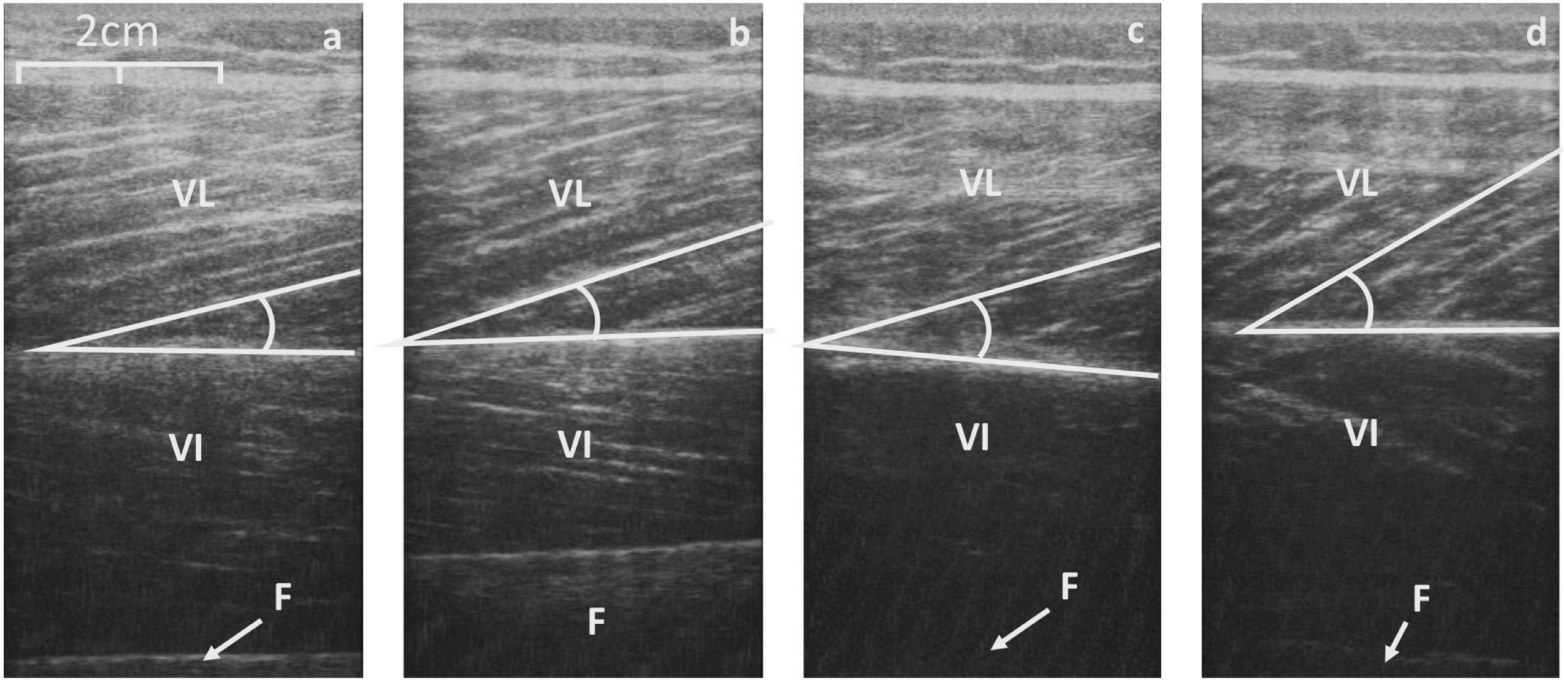
Sagittal scans of the VL at rest (**a**, control; **c**, Achondroplasia) and at iMVC (**b**, control; **d**, Achondroplasia). Deep aponeurosis is highlighted along with a fascicular insertion. VL, Vastus Lateralis; VI, Vastus Intermedius; F, Femur.

There was a significant effect in VL fiber length from rest to iMVC [*F*_(1, 25)_ = 69.65, *P* < 0.001, η^2^ = 72.8] and a between group effect [*F*_(1, 25)_ = 4.260, *P* = 0.048, η^2^ = 12.9], but no interaction was observed [*F*_(1, 25)_ = 0.545, *P* = 0.467, η^2^ = 2.1]. Similarly, there was a significant effect in VL pennation angle from rest to iMVC [*F*_(1, 25)_ = 68.28, *P* < 0.001, η^2^ = 72.4] and a between group effect [*F*_(1, 25)_ = 6.191, *P* = 0.020, η^2^ = 19.2], but no interaction was observed [*F*_(1, 25)_ = 2.645, *P* = 0.116, η^2^ = 9.2]. There was no difference in VL pennation angle (*P* = 0.105) or fascicle length (*P* = 0.199) at rest between groups, but Achondroplasic fascicles shortened 28% more (*P* = 0.012) and increased by 25% in pennation angle (*P* = 0.029) from rest to iMVC compared to controls (Figures 3, 4). The Achondroplasic group also had a 36% longer fascicle length relative to VL length (*P* < 0.001, Table 1).

### Patella tendon force-elongation relationship

**Figure 5:**
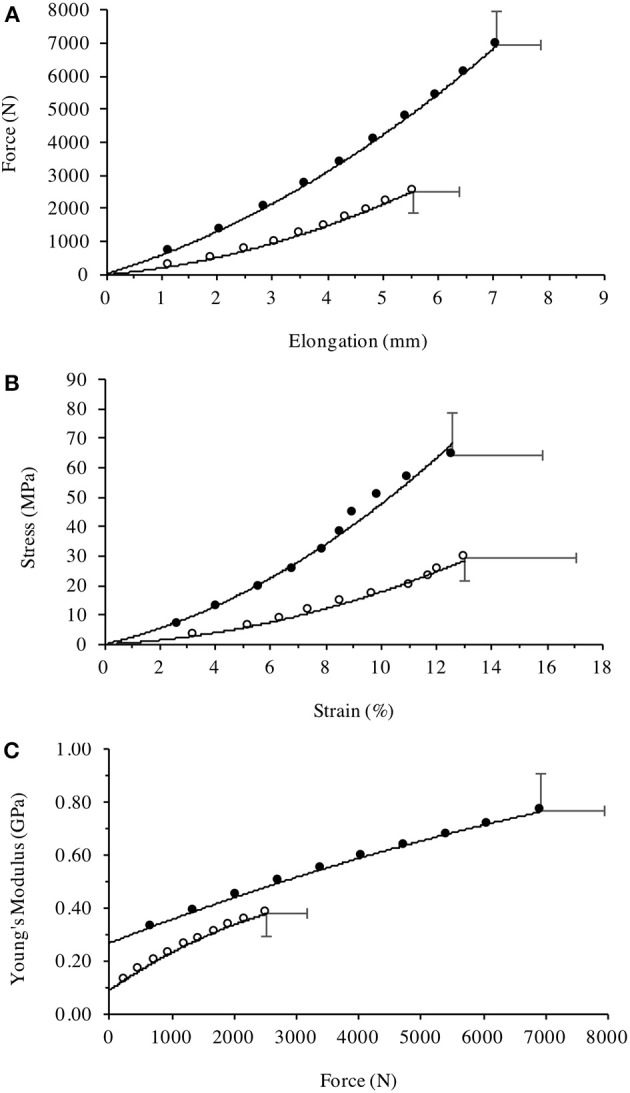
Patella tendon **(A)** Force-Elongation relationship, **(B)** stress-strain relationship, and **(C)** Young's modulus plotted against the incremental level of iMVC force for the Achondroplasic (open circles) and control group (closed circles). All figures are fitted with polynomial curves, with Figures 4a, b forced through zero. For clarity of Figures, SD are only given at max values.

The Achondroplasic group produced less F_PT_ than controls [*F*_(9, 216)_ = 71.85, *P* < 0.001, η^2^ = 75.0], with an average of 63% less F_PT_ at each 10% interval (*P* < 0.001) and 64% less maximal F_PT_ than controls (*P* < 0.001, *d* = 5.315, Figure 5A). The Achondroplasic tendon elongation was less than controls [*F*_(9, 216)_ = 32.948, *P* < 0.001, η^2^ = 57.9] with an average 15% less elongation compared to controls at each 10% interval (each 10% interval: *P* < 0.001) and 22% less at KE iMVC compared to controls (*P* < 0.001, *d* = 3.837, Figure 5A).

### Tendon stress-strain

Patella tendon strain was similar between groups [*F*_(9, 216)_ = 0.376, *P* = 0.545, η^2^ = 1.5] during KE with maximal strain being 13 (4%) and 13 (3%) for the Achondroplasic and control group, respectively (Figure 5B). Stress was lower however, in the Achondroplasic group during KE [*F*_(9, 216)_ = 43.81, *P* < 0.001, η^2^ = 64.6], with an average 55% less patella tendon stress at each 10% interval (each 10% interval: *P* < 0.001) and 53% lower maximal patella tendon stress compared to controls (*P* < 0.001, *d* = 3.191, Figure 5B).

### Tendon stiffness

Patella Tendon stiffness was lower in the Achondroplasic group [*F*_(9, 216)_ = 106.8, *P* < 0.001, η^2^ = 81.6] with patella tendon stiffness being on average 51% lower through the 10–90% intervals of KE iMVC compared to controls (each 10% interval: *P* < 0.01). The Achondroplasic group had a 47% lower maximal patella tendon stiffness compared to controls (Achondroplasic group, 748 N·mm^-1^ (93); controls, 1418 N·mm^-1^ (101), *P* < 0.001, *d* = 6.890).

### Young's modulus

Young's Modulus was also lower in the Achondroplasic group compared to controls [*F*_(9, 216)_ = 74.21, *P* < 0.001, η^2^ = 74.8]. Young's Modulus was on average 54% lower in Achondroplasic group at each 10% of KE iMVC (each 10% interval: *P* < 0.001, Figure 5C) and was 51% lower at KE iMVC than controls (*P* < 0.001, *d* = 3.465, Figure 5C).

### Standardized tendon properties

Tensile properties of all participants were analyzed at 1756 N (the F_PT_ attained by the weakest Achondroplasic participant). Consistent with absolute measures of F_PT_, stress (22%, *P* = 0.001) and strain (78%, *P* < 0.001) were higher in the Achondroplasic group, while their stiffness (12%, *P* < 0.001) and Young's Modulus (11%, *P* = 0.041) were lower compared to controls (Table 3).

**Table 3:** Elastic properties of Achondroplasic and control patella tendon at a standardized force (1756 N).

	**Achondroplasia**	**Control**	***P*-value**	**Test statistic**	**Effect size**	**Mean difference**	**95% CI Lower**	**95% CIUpper**
Stress (MPa)	20.9 (3.2)	16.4 (2.8)	0.001	3.869	1.516	4.5	2.1	6.9
Strain (%)	11.2 (3.8)	2.5 (2.0)	< 0.001	5.842	2.325	0.1	0.0	0.1
Stiffness (N·mm^-1^)	637 (39)	721 (10)	< 0.001	8.750	3.562	83	64	103
Young's Modulus (GPa)	0.32 (0.06)	0.36 (0.06)	0.041	1.812	0.696	0.04	-0.01	0.09

*Values displayed as mean (SD)*.

## Discussion

The aim of this study was to assess the *in vivo* morphological and material properties of Achondroplasic patella tendons during KE iMVC and compare them to controls. The Achondroplasic patella tendon was smaller in CSA and volume but were disproportionate in length compared to controls. The main finding however, was that despite accounting for patella tendon morphology and mechanical properties, the tensile strength (here as Young's Modulus) of the Achondroplasic patella tendon were significantly lower than controls' leading to a more compliant Achondroplasic patella tendon.

### Patella tendon morphology and mechanical properties

Many agree that a larger ratio of tendon CSA to tendon length leads to a greater tendon stiffness (Kubo et al., 2001b; Reeves, 2006; O'Brien et al., 2010b). While this ratio was higher in the Achondroplasic patella tendon, a higher patella tendon stiffness was not observed. A shorter Achondroplasic femur length compared to controls (measured here as VL length) would explain the difference in Achondroplasic patella tendon length. A shorter femur would suggest a shorter muscle tendon unit, which is observed elsewhere in groups of shorter statures (Morse et al., 2008; O'Brien et al., 2010a,b). However, the shorter femur observed in the Achondroplasic group compared to controls does not explain the Achondroplasic group's relatively longer patella tendon length. The Achondroplasic knee is more flexed when at rest (Akyol et al., 2015), and the more flexed knee may deform the patella tendon over time leading to a creep effect. However, no longitudinal measures of the Achondroplasic tendon has been made.

With no *in vitro* tendon properties identified in the Achondroplasic population, explanations as to why absolute measures of CSA_PT_ are lower and relative measures (CSA_PT_ to patella tendon length) are higher compared to controls are speculative, but reasonable. The measurement of CSA_PT_ using ultrasound is regarded by some, though by few, as unreliable (Ekizos et al., 2013). However, the present data, previous reports from our own lab (Reeves et al., 2003b), and recently by Gellhorn and Carlson (2013), show high ICCs for CSA_PT_ with a typical error of < 1.5 mm^2^. Using the maximal CSA_PT_ difference from Reeves et al., the ranges of calculated stress measured here would be 3.6 and 2.8% different for the Achondroplasic and control group, respectively. Even the lower ICCs presented by Ekizos et al. would present stress values ±20% of the average values for each of the current groups. If the measurement of CSA_PT_ was indeed miscalculated in this study, it would have to have been ~40% under- and over-predicted for the Achondroplasic and control group, respectively, for there to be the same stress and therefore the same stiffness values.

The use of ultrasound is a good method of attaining CSA_PT_ without the use and availability of a magnetic resonance imaging (MRI) scanner, which is considerably less accessible for most researchers. Despite the conflicting arguments in the reliability of CSA_PT_ calculation, the stress and Young's Modulus calculated here have a large amount of face validity. These results are undoubtedly due to the large differences in relative (CSA_PT_ to patella tendon length) and absolute CSA_PT_ between groups. Importantly though, this is backed by the potential respective under- and over-predictions of the Achondroplasic and control groups' CSA_PT_ required to attain similar stress values. Therefore, the smaller absolute CSA_PT_ seen in the Achondroplasic group would most likely be a result of the scaling of leg length and muscle size. The larger relative CSA_PT_ in the Achondroplasic group is most probably “pseudohypertrophy” of the tendon fibrils and is likely due to intrinsic factors of the tendon, which are discussed later.

We have previously observed that Achondroplasic adults have a lower net KE iMVCτ than controls (Sims et al., 2017). This in turn led to a lower F_PT_ and patella tendon stress in the Achondroplasic group. Initially this would have been a viable reason to explain the Achondroplasic group's lower patella tendon Young's Modulus, particularly as patella tendon strain was similar between groups at iMVC. However, to observe the mechanical properties of the patella tendon between groups, we standardized F_PT_ between groups. Stress and strain were higher in the Achondroplasic group while their stiffness and Young's Modulus was lower than controls. This finding is different to other reports where Young's Modulus at iMVC is different but are similar when calculated at a standardized tendon forces (Onambélé et al., 2007; Burgess et al., 2009). The lower Young's Modulus at this standardized value again suggest that the intrinsic properties of the Achondroplasic patella tendon are different to controls.

### Young's modulus differences

In the present study, the higher Achondroplasic Young's Modulus at maximal and standardized F_PT_ suggests that the Achondroplasic patella tendon's tensile properties are different to controls. The Young's Modulus of the Achondroplasic patella tendon appears to be lower than adult females (Onambélé et al., 2007), children of both genders (O'Brien et al., 2010b), the elderly (Reeves et al., 2003b) and a large number of controls groups that are similar in age, stature as the control group presented here (Onambélé et al., 2007; Carroll et al., 2008; Seynnes et al., 2009; O'Brien et al., 2010b; Hicks et al., 2017). There appears to be no conclusive evidence to suggest why a lower Young's Modulus exists in the Achondroplasic group, however there are a number of speculative but reasonable explanations as to why Achondroplasic patella tendons would be more compliant than controls. Whilst this list is not exhaustive, the mutation that causes Achondroplasia, the tendon's extracellular matrix, hormonal differences and body composition of Achondroplasic individuals may all contribute to a more compliant Achondroplasic patella tendon. For example, the Achondroplasic male adults adiposity and body mass index are higher than controls (Sims et al., 2017), and there are known genetic mutations of collagen formation within Achondroplasia, symptomatic of the condition (Benjamin and Ralphs, 2000; Horton et al., 2007). Both factors may play a role in the development and orientation of the patella tendon type II collagen in the fibrocartilage of FGFR3 mutated mice which is negatively affected (Liang et al., 2009). Were fibrocartilage to be similarly affected by FGFR3 in humans, the compliance of the fibrocartilage may be reduced. Furthermore, high adiposity has previously been associated with hormonal differences (Miller et al., 2007; Hansen et al., 2009) and higher tendon water content (Laaksonen et al., 2003), which are in turn linked to more compliant tendons (Kubo et al., 2001b; Birch, 2007; Onambélé et al., 2007). As mentioned however, these factors are speculative and further *in vivo* and *in vitro* investigations are warranted.

### Clinical implications

A more compliant tendon alters the length tension relationship whereby the muscle fascicle works on the ascending limb of the relationship (Reeves, 2006). This observation may partly explain the lower specific force production of the Achondroplasic VL (Sims et al., 2017). Furthermore, a more compliant tendon will undergo a greater excursion for the same force, as observed within our Achondroplasic group at a standardized force value (1756 N). The higher Achondroplasic patella tendon compliance is likely to affect postural balance in Achondroplasic populations, as observed in control populations (Onambélé et al., 2006). This would suggest that Achondroplasic individuals are at a greater risk of falling compared to controls. Furthermore, compliant tendons are associated with a higher metabolic cost during gait (Arampatzis et al., 2006; Fletcher et al., 2010; Albracht and Arampatzis, 2013) and amplification of muscle power during muscle contraction (Roberts and Azizi, 2011). We have recently showed both of these artifacts in Achondroplasic males with the population having a higher walking and running metabolic cost (Sims et al., 2018) and a lower specific VL force production compared to controls (Sims et al., 2017). The more compliant Achondroplasic patella tendon observed in the current study would suggest plausible reasoning for their higher metabolic cost and lower amplification of force production from the muscle. Therefore, improvements of Achondroplasic tendon compliance through resistance training would likely improve their higher metabolic cost, as observed in controls (Fletcher et al., 2010). To the authors' knowledge though, no such training study in Achondroplasic individuals exist.

Although there is a potential increase in injury risk to Achondroplasic tendons, research groups have shown that tendons are adaptable structures which can increase in stiffness when put through a resistance training programme and therefore reduce the risk of injury and improve metabolic cost. Such programmes are observed in controls (Kubo et al., 2001a,b; Onambélé et al., 2008; Seynnes et al., 2009), the elderly (Reeves et al., 2003a,b; Onambélé et al., 2006, 2008) and children (Waugh et al., 2014). The likely lower risk of falling and a lower metabolic cost following such programmes in physically active, sedentary and elderly Achondroplasic groups would possibly improve the prevalence at which they effectively complete activities of daily living and physical activity. This in turn would likely have positive effects on their health (Wilmot et al., 2012). Future work should therefore be aimed to chronicle the effects of differing training regimens in Achondroplasic populations, specifically observing the changing of gait related tendon compliance, such as the patella and Achilles tendon.

### Limitations

To the authors' knowledge, this paper presents the first data regarding the *in vivo* mechanical properties of the Achondroplasic patella tendon. There are however, a number of limitations to the methods employed that warrant further development to confirm the presented data. The quantification of the Achondroplasic and control group's CSA_PT_ was through ultrasonography. While ultrasound to quantify CSA_PT_ is considered reliable (Mc Auliffe et al., 2017) and used in numerous populations (Reeves et al., 2003b; Onambélé et al., 2007; Burgess et al., 2009; Gellhorn and Carlson, 2013), the technique has not been validated against MRI. It should be noted however that our control group's CSA_PT_ is similar to that presented previously using both ultrasonography (Onambélé et al., 2007; O'Brien et al., 2010b) and MRI (Carroll et al., 2008; Seynnes et al., 2009). We are confident therefore, that the large effect sizes observed between the current groups' CSA_PT_ would be persist despite the likely higher variance in measurement when using ultrasonography compared to MRI.

Net KE iMVCτ was derived by the measurement of KF coactivation during KE iMVC on an isokinetic dynamometer. Both the methods of correcting for coactivation and the use of an isokinetic dynamometer could be considered to be a limitation to the calculation of net KE iMVCτ and F_PT_, respectively. Based on previous data (Macaluso et al., 2002), there is 5% KE coactivation during KF iMVC, this is equivalent to 13 N·m in controls and 8 N·m in the Achondroplasic group, assuming KE coactivation is three times greater like that observed in Achondroplasic KF coactivation. By omitting the calculation of KE coactivation during our measurement of KF iMVC, we underestimated the KF iMVCτ and have therefore underestimated our net KE iMVCτ. It should be noted however, that because KE coactivation is very low during KF, this level of underestimation of coactivation torque would increase KE iMVCτ by 12% in the Achondroplasic group and 5% in controls. In the present study, by making this correction, the group differences in net KE iMVCτ would be 61% rather than the 63% reported. Due to the circular nature of accounting for coactivation in this manner however, we have only corrected net KE iMVCτ calculations based on the much higher coactivation of the KF during KE iMVC.

The use of a dynamometer, although ubiquitous within research observing neuromuscular and biomechanical properties of the musculotendon unit during iMVCs, is likely to result in a degree of change of the knee joint angle that could contribute to errors in torque measurement. At the level of net KE iMVCτ observed in the present controls group, a prediction of unmeasured torque can be made using the change in the knee joint angle from 90° to ~70° (Tsaopoulos et al., 2011). At this level of joint displacement (20°), we would expect a lengthening of the patella tendon moment arm by 3.3 mm (Baltzopoulos, 1995), although a lower iMVC was seen in the Achondroplasic group which would suggest a lower joint displacement during at iMVC (Kaufman et al., 1995; Arampatzis et al., 2004; Tsaopoulos et al., 2011). Assuming the same level of moment arm lengthening in both groups (3.3 mm), the F_PT_ would reduce by 8% in the Achondroplasic group and 7% in the controls. As these estimates of joint deformation as a result of dynamometer compliance are unverified, we present the unaltered values as to allow comparison to related work.

## Conclusion

This study aimed to examine the *in vivo* material properties of adult Achondroplasic patella tendon during a ramped MVC. The main findings are that there are significant differences in material properties between Achondroplasic males and controls, which lead to a more compliant Achondroplasic patella tendon. In addition, at both absolute (iMVC) and standardized force (1756 N) levels of stress, stiffness and Young's modulus are lower in the Achondroplasic groups compared to controls suggesting intrinsic differences between groups.

## New and noteworthy

Achondroplasic patella tendon morphology and mechanical properties are different to controls leading to a more compliant patella tendon during maximal voluntary contraction. The genetic variation that alters collagen development in the bone and causes Achondroplasia, may also affect their tendon compliance.

## Ethics statement

This study was carried out in accordance with the recommendations of Manchester Metropolitan Universities locale this committee with written informed consent from all subjects. All subjects gave written informed consent in accordance with the Declaration of Helsinki.

## Author contributions

DS lead author, contributed to the inception of the study, participant recruitment and testing, data analysis and interpretation, writing of the main body of work and approval of the article. GO-P and AB contributed to the inception of the study, data analysis and interpretation and revisions of the main body of work and approval of the article. CP contributed to data analysis and interpretation and revisions of the main body of work and approval of the article. CM contributed to the inception of the study, participant recruitment and testing, data analysis and interpretation and writing of the main body of work and approval of the article.

### Conflict of interest statement

The authors declare that the research was conducted in the absence of any commercial or financial relationships that could be construed as a potential conflict of interest.
