# Supplementary material for: Morphological and Mechanical Properties of the Human Patella Tendon in Adult Males With Achondroplasia
Source: Front Physiol. 2018 Jul 20;9:867. doi: 10.3389/fphys.2018.00867 (PMC6063341; doi:10.3389/fphys.2018.00867)
Supplement: Supplementary file 1 [file Table_1.pdf]

Supplementary data for the following publication: Sims et al. (2018) 'Morphological and mechanical properties of the human patella tendon in adult males with Achondroplasia.' *Frontiers in Physiology.*

Table 1a) Main effect statistics for torque production during isometric volentary contraction of the knee extensors in adult males with Achondroplasia and controls.

	df	F Ratio	P Value	η^2
Within	9,216	35.724	< 0.001	93.7
Between	1,24	63.171	< 0.001	72.5
Interaction	9,216	62.946	< 0.001	72.4

df, Degrees of Freedom; η^2, Partial eta squared

Table 1b) Between group post hoc comparisons of torque production (N·m) of the knee extensors at 10% intervals of isometric maximal volentary contraction in Achondroplasic males and controls. Values given as mean (SD).

% Max	Achondroplasia	Control	P Value	Effect Size	Mean Diff.	95% CI Low	95% CI Upp
10	8.9 (1.9)	25.4 (3.8)	< 0.001	5.781	16.5	11.2	19.0
20	17.8 (3.9)	50.8 (7.5)	< 0.001	5.781	33.0	22.3	38.0
30	26.7 (5.8)	76.2 (11.3)	< 0.001	5.781	49.5	33.5	56.9
40	35.6 (7.7)	101.6 (15.1)	< 0.001	5.781	65.9	44.6	75.9
50	44.6 (9.7)	127.0 (18.9)	< 0.001	5.781	82.4	55.8	94.9
60	53.5 (11.6)	152.4 (22.6)	< 0.001	5.781	98.9	66.9	113.9
70	62.4 (13.5)	177.8 (26.4)	< 0.001	5.781	115.4	78.1	132.9
80	71.3 (15.4)	203.2 (30.2)	< 0.001	5.781	131.9	89.2	151.8
90	80.2 (17.4)	227.9 (34.6)	< 0.001	5.678	147.7	99.3	170.4
100	92.8 (20.0)	260.1 (37.9)	< 0.001	5.772	167.3	113.9	192.6

Mean Diff., Mean Difference; CI, Confidence Interval of the Mean Difference.

Table 2a) Main effect statistics for patella tendon elongation during isometric volentary contraction of the knee extensors in adult males with Achondroplasia and controls.

	df	F Ratio	P Value	η^2
Within	9,216	102.091	< 0.001	81
Between	1,24	32.948	< 0.001	57.9
Interaction	9,216	2.65	< 0.001	9.9

df, Degrees of Freedom; η^2, Partial eta squared

Table 2b) Between group post hoc comparisons of patella tendon elongation (mm) at 10% intervals of isometric maximal volentary knee extensor contraction in Achondroplasic males and controls. Values given as mean (SD).

% Max	Achondroplasia	Control	P Value	Effect Size	Mean Diff.	95% CI Low	95% CI Upp
10	38.3 (3.9)	52.4 (4.3)	< 0.001	3.429	14.1	7.8	16.6
20	39.1 (3.8)	53.2 (4.8)	< 0.001	3.289	14.1	7.5	16.8
30	39.5 (3.5)	54.1 (4.6)	< 0.001	3.582	14.5	8.0	17.0
40	40.0 (3.6)	54.8 (4.8)	< 0.001	3.534	14.8	8.1	17.5
50	40.5 (3.4)	55.4 (4.8)	< 0.001	3.632	15.0	8.2	17.6
60	41.0 (3.5)	55.8 (4.9)	< 0.001	3.557	14.8	8.3	17.7
70	41.6 (3.1)	56.1 (5.0)	< 0.001	3.580	14.5	8.0	17.3
80	41.9 (3.3)	56.7 (5.1)	< 0.001	3.541	14.7	8.0	17.5
90	42.1 (3.5)	57.4 (5.0)	< 0.001	3.594	15.3	8.5	18.0
100	42.5 (3.4)	58.4 (4.9)	< 0.001	3.837	15.8	9.3	18.7

Mean Diff., Mean Difference; CI, Confidence Interval of the Mean Difference.

Table 3a) Main effect statistics for patella tendon elongation during isometric volentary contraction of the knee extensors in adult males with Achondroplasia and controls.

	df	F Ratio	P Value	η^2
Within	9,216	397.064	< 0.001	94.3
Between	1,24	71.85	< 0.001	75
Interaction	9,216	71.081	< 0.001	74.8

df, Degrees of Freedom; η^2, Partial eta squared

Table 3b) Between group post hoc comparisons of patella tendon force (N) at 10% intervals of isometric maximal volentary knee extensor contraction in Achondroplasic males and controls. Values given as mean (SD).

% Max	Achondroplasia	Control	P Value	Effect Size	Mean Diff.	95% CI Low	95% CI Upp
10	241 (62)	677 (99)	< 0.001	5.411	436	307	504
20	482 (125)	1353 (197)	< 0.001	5.411	871	614	1009
30	723 (187)	2030 (296)	< 0.001	5.411	1307	921	1513
40	964 (250)	2707 (394)	< 0.001	5.411	1743	1228	2017
50	1205 (312)	3384 (493)	< 0.001	5.411	2178	1535	2521
60	1446 (375)	4060 (591)	< 0.001	5.411	2614	1842	3026
70	1687 (437)	4737 (690)	< 0.001	5.411	3050	2148	3530
80	1928 (500)	5414 (789)	< 0.001	5.411	3485	2455	4034
90	2169 (562)	6071 (904)	< 0.001	5.325	3902	2734	4526
100	2511 (659)	6931 (1005)	< 0.001	5.315	4420	3125	5123

Mean Diff., Mean Difference; CI, Confidence Interval of the Mean Difference.

Table 4a) Main effect statistics for patella tendon strain during isometric volentary contraction of the knee extensors in adult males with Achondroplasia and controls.

	df	F Ratio	P Value	η^2
Within	9,216	89.676	< 0.001	78.9
Between	1,24	0.376	0.545	1.5
Interaction	9,216	0.564	0.826	2.3

df, Degrees of Freedom; η^2, Partial eta squared

Table 4b) Between group post hoc comparisons of patella tendon strain (%) at 10% intervals of isometric maximal volentary knee extensor contraction in Achondroplasic males and controls. Values given as mean (SD).

% Max	Achondroplasia	Control	P Value	Effect Size	Mean Diff.	95% CI Low	95% CI Upp
10	3.3 (2.3)	2.7 (1.5)	0.612	0.311	31.1	-1.9	1.2
20	5.2 (3.5)	4.0 (2.5)	0.385	0.393	39.3	-3.5	1.4
30	6.3 (3.2)	5.6 (2.8)	0.550	0.242	24.2	-3.1	1.7
40	7.4 (3.2)	6.8 (3.0)	0.764	0.190	19.0	-3.0	2.2
50	8.5 (3.7)	7.9 (3.0)	0.740	0.191	19.1	-3.3	2.4
60	9.7 (3.4)	8.5 (3.3)	0.627	0.344	34.4	-3.5	2.2
70	11.0 (4.1)	9.0 (3.2)	0.299	0.570	57.0	-4.6	1.5
80	11.7 (3.9)	9.9 (3.3)	0.398	0.507	50.7	-4.3	1.8
90	12.0 (3.6)	11.0 (3.4)	0.588	0.298	29.8	-3.8	2.2
100	13.0 (4.1)	12.6 (3.3)	0.934	0.114	11.4	-3.0	3.2

Mean Diff., Mean Difference; CI, Confidence Interval of the Mean Difference.

Table 5a) Main effect statistics for patella tendon stress during isometric volentary contraction of the knee extensors in adult males with Achondroplasia and controls.

	df	F Ratio	P Value	η^2
Within	9,216	3433.253	< 0.001	93.5
Between	1,24	43.808	< 0.001	64.6
Interaction	9,216	43.982	< 0.001	64.7

df, Degrees of Freedom; η^2, Partial eta squared

Table 5b) Between group post hoc comparisons of patella tendon stress (MPa) at 10% intervals of isometric maximal volentary knee extensor contraction in Achondroplasic males and controls. Values given as mean (SD).

% Max	Achondroplasia	Control	P Value	Effect Size	Mean Diff.	95% CI Low	95% CI Upp
10	2.8 (0.8)	6.3 (1.4)	< 0.001	3.209	3.5	2.3	4.5
20	5.6 (1.5)	12.6 (2.8)	< 0.001	3.209	7.0	4.7	8.9
30	8.5 (2.3)	18.9 (4.3)	< 0.001	3.209	10.5	7.0	13.4
40	11.3 (3.0)	25.2 (5.7)	< 0.001	3.209	14.0	9.3	17.9
50	14.1 (3.8)	31.5 (7.1)	< 0.001	3.209	17.5	11.7	22.3
60	16.9 (4.5)	37.9 (8.5)	< 0.001	3.209	20.9	14.0	26.8
70	19.7 (5.3)	44.2 (9.9)	< 0.001	3.209	24.4	16.3	31.2
80	22.5 (6.0)	50.5 (11.4)	< 0.001	3.209	27.9	18.7	35.7
90	25.4 (6.8)	56.6 (12.7)	< 0.001	3.202	31.2	20.8	39.9
100	29.4 (8.0)	64.5 (14.0)	< 0.001	3.191	35.2	23.8	44.9

Mean Diff., Mean Difference; CI, Confidence Interval of the Mean Difference.

Table 6a) Main effect statistics for patella tendon stiffness during isometric volentary contraction of the knee extensors in adult males with Achondroplasia and controls.

	df	F Ratio	P Value	η^2
Within	9,216	1134.06	< 0.001	97.9
Between	1,24	106.768	< 0.001	81.6
Interaction	9,216	52.333	< 0.001	68.6

df, Degrees of Freedom; η^2, Partial eta squared

Table 6b) Between group post hoc comparisons of patella tendon stiffness ($N \cdot mm^{-1}$) at 10% intervals of isometric maximal volentary knee extensor contraction in Achondroplasic males and controls. Values given as mean (SD).

% Max	Achondroplasia	Control	P Value	Effect Size	Mean Diff.	95% CI Low	95% CI Upp
10	271 (24)	668 (19)	< 0.001	18.329	397	295	427
20	354 (37)	786 (32)	< 0.001	12.434	432	321	470
30	420 (47)	888 (43)	< 0.001	10.411	467	346	512
40	478 (55)	979 (51)	< 0.001	9.384	502	371	552
50	529 (63)	1063 (59)	< 0.001	8.762	534	394	590
60	576 (69)	1141 (66)	< 0.001	8.349	565	416	626
70	619 (75)	1213 (73)	< 0.001	8.053	594	437	660
80	659 (80)	1281 (79)	< 0.001	7.810	623	457	693
90	697 (86)	1345 (86)	< 0.001	7.548	648	474	723
100	748 (93)	1418 (101)	< 0.001	6.890	670	486	753

Mean Diff., Mean Difference; CI, Confidence Interval of the Mean Difference.

Table 7a) Main effect statistics for patella tendon Young's Modulus during isometric volentary contraction of the knee extensors in adult males with Achondroplasia and controls.

	df	F Ratio	P Value	η^2
Within	9,216	487.7	< 0.001	95.1
Between	1,24	74.21	< 0.001	74.8
Interaction	9,216	37.136	< 0.001	59.8

df, Degrees of Freedom; η^2, Partial eta squared

Table 7b) Between group post hoc comparisons of patella tendon Young's Modulus (GPa) at 10% intervals of isometric maximal volentary knee extensor contraction in Achondroplasic males and controls. Values given as mean (SD).

% Max	Achondroplasia	Control	P Value	Effect Size	Mean Diff.	95% CI Low	95% CI Upp
10	0.12 (0.03)	0.32 (0.05)	< 0.001	4.756	0.20	0.16	0.24
20	0.17 (0.04)	0.39 (0.07)	< 0.001	4.049	0.22	0.17	0.27
30	0.20 (0.05)	0.45 (0.08)	< 0.001	3.877	0.25	0.19	0.30
40	0.23 (0.06)	0.50 (0.09)	< 0.001	3.718	0.27	0.20	0.33
50	0.26 (0.06)	0.55 (0.10)	< 0.001	3.648	0.29	0.22	0.36
60	0.28 (0.07)	0.59 (0.10)	< 0.001	3.540	0.31	0.23	0.39
70	0.31 (0.07)	0.63 (0.11)	< 0.001	3.487	0.32	0.24	0.41
80	0.33 (0.08)	0.67 (0.12)	< 0.001	3.472	0.34	0.26	0.43
90	0.35 (0.08)	0.72 (0.12)	< 0.001	3.529	0.37	0.28	0.46
100	0.38 (0.09)	0.77 (0.14)	< 0.001	3.465	0.39	0.29	0.49

Mean Diff., Mean Difference; CI, Confidence Interval of the Mean Difference.

Table 8a) Between group post hoc comparisons of vastus lateralis pennation angle (°) and fibre length (cm) from rest to isometric volentary contraction in adult males with Achondroplasia and controls. Values displayed as mean (SD).

	Achondroplasia	Control	P Value	Effect Size	Mean Diff.	95% CI Low	95% CI Upp
Pennation at Rest	15.6 (3.2)	13.8 (2.4)	0.105	0.645	1.801	-0.402	4.005
Pennation at iMVC	20.9 (4.6)	17.4 (2.4)	0.012	1.013	3.541	0.839	6.243
Fibre Length at Rest	9.5 (2.4)	10.5 (1.5)	0.199	0.495	0.969	-0.542	2.479
Fibre Length at iMVC	6.8 (1.5)	8.2 (1.5)	0.029	0.926	1.408	0.16	2.656

iMVC, isometric Maximal Volentary Contraction; CI, Confidence Interval of the Mean Difference

Table 8b) Within group post hoc comparisons of vastus lateralis pennation angle (°) from rest to isometric volentary contraction in adult males with Achondroplasia and controls. Values displayed as mean (SD).

	Pennation Rest	Pennation MVC	P Value	Effect Size	Mean Diff.	95% CI Low	95% CI Upp
Achondroplasia	15.6 (3.2)	20.9 (4.6)	< 0.001	1.361	3.55	2.236	4.864
Control	13.8 (2.4)	17.4 (2.4)	< 0.001	1.467	5.29	3.527	7.053

Mean Diff., Mean Difference; CI, Confidence Interval of the Mean Difference.

Table 8c) Within group post hoc comparisons of vastus lateralis fibre length (cm) from rest to isometric volentary contraction in adult males with Achondroplasia and controls. Values displayed as mean (SD).

	Fibre Length Rest	Fibre Length MVC	P Value	Effect Size	Mean Diff.	95% CI Low	95% CI Upp
Achondroplasia	9.5 (2.4)	6.8 (1.5)	< 0.001	1.367	2.7	1.72	3.68
Control	10.5 (1.5)	8.2 (1.5)	< 0.001	1.489	2.261	1.531	2.991

Mean Diff., Mean Difference; CI, Confidence Interval of the Mean Difference.
